# The ubiquitin ligase RNF181 stabilizes ERα and modulates breast cancer progression

**DOI:** 10.1038/s41388-020-01464-z

**Published:** 2020-09-24

**Authors:** Jian Zhu, Xin Li, Peng Su, Min Xue, Yifeng Zang, Yinlu Ding

**Affiliations:** 1 Department of general surgery, the Second Hospital, Cheeloo College of Medicine, Shandong University, 250033 Jinan, China; 2 Xinxiang Key Laboratory of Tumor Migration, Invasion and Precision Medicine, School of Laboratory Medicine, Henan Collaborative Innovation Center of Molecular Diagnosis and Laboratory Medicine, Xinxiang Medical University, Xinxiang, 453003, Henan Province P.R. China; 3 Department of Pathology, Qilu Hospital, Cheeloo College of Medicine, Shandong University, 250033 Jinan, China

**Keywords:** Breast cancer, Hormone receptors

## Abstract

ERα positive breast cancer accounts for 70% of breast malignancies. Compared with ERα negative types, ERα positive breast cancer could be effective controlled by endocrine therapy. However, more than half of the patients will develop endocrine resistance, making it an important clinical issue for breast cancer therapy. Endocrine resistance might be caused by multiple alternations, including the components of ERα signaling, during tumor progression. Thus, it is urgent and necessary to uncover the molecular mechanisms that controls ERα expression and stability to improve breast cancer therapeutics. In our current study, we identifies that the ubiquitin ligase RNF181 stabilizes ERα and facilitates breast cancer progression. The expression of RNF181 is correlated with ERα level in human breast tumors and relates to poor survival in endocrine-treated patients. RNF181 depletion inhibits breast cancer progression in vivo and in vitro, reduces ERα protein level and its target gene expression, such as PS2 and GREB1. Unbiased RNA sequencing analysis indicates RNF181 is necessary for ERα signature gene expression in whole genomic level. Immuno-precipitation assays indicate that RNF181 associates with ERα and promotes its stability possibly via inducing ERα K63-linked poly-ubiquitination. In conclusion, our data implicate a non-genomic mechanism by RNF181 via stabilizing ERα protein controls ERα target gene expression linked to breast cancer progression.

## Introduction

Breast cancer is the most common female malignancy worldwide [1]. According to latest cancer epidemiological report, more than 1.6 million breast cancer cases are diagnosed each year, which account for about 20% of all women malignancies [2]. Based on the clinical-pathological classification, breast cancers are grouped as Endocrine receptor-positive type (positive for estrogen receptors or progesterone receptors), HER2-positive type, and triple negative type (TBNC) (negative for estrogen receptors, progesterone receptors, and HER2) [3]. Compared with HER2 positive and triple negative breast cancer subtypes, ERα positive breast cancer patients show a significant priority in prognosis and could benefit from endocrine therapy [4]. However, approximately half of the patients will develop endocrine resistance overtime, making it a major challenge for both clinics and basic researches [5–7]. Thus, the insight into the detailed mechanisms, which control the ERα signaling activity, is critical for developing novel therapeutics for breast cancer.

The relation between ERα and breast cancer was discovered 30 years ago [8]. ERα belongs to the member of the nuclear receptor superfamily and composed of three functional domains: a DNA-binding domain (DBD), a ligand-binding domain (LBD) and one transcriptional activation domain (AF1) [9]. When ERα is activated by estrogen, it trans-locates into the nucleus, forms the hemo-dimers and binds to the promoter regions of ERα target genes, which subsequently promotes breast cancer initiation and progression. Clinically, ERα expression level correlates with breast cancer risk and two-thirds of breast cancers have elevated level of ERα. The selective estrogen receptor modulators, such as tamoxifen, share the structure similarity with estradiol, which bind to ERα protein and block its transcriptional activity in breast cancer [10, 11].

Although there are several possible and confirmed mechanisms for endocrine resistance, the detailed mechanisms are still not clear. Besides a small proportion of breast cancers, which lose ERα expression during endocrine therapy, most of endocrine resistant breast tumors still maintain ERα expression [12]. Several studies pointed out that the co-activators of ERα signaling coupled with ERα modifications contribute to enhanced ERα signaling and endocrine resistance [13–15]. Thus, understanding the ubiquitin-proteasome system of ERα protein, including numerous E3 ubiquitin ligases, coupled with modulating ERα protein stability could be a promising strategy for breast cancer therapeutics, especially for endocrine resistant patients.

There are approximately more than 700 E3 ubiquitin ligases, which could be grouped into four families according to the functional domains: HECT type, RING type, U-box type, and PHD-finger type [16]. Among these groups, the RING finger E3 ligase group is the biggest, while the function is largely unknown. Our previous studies identified several RING proteins, which were elevated in breast cancer and modulates cancer progression [17, 18]. In our current study, we implicate RNF181 (RING Finger Protein 181) in modulating ERα protein stability and breast cancer progression. RNF181 associates with AF1 domain of ERα via its RING domain and prolongs ERα stability, which subsequently enhances ERα target gene expression and breast cancer cell proliferation.

## Results

### **RNF181 is elevated in breast cancer and correlates with poor survival in endocrine therapy patients**

**Fig. 1:**
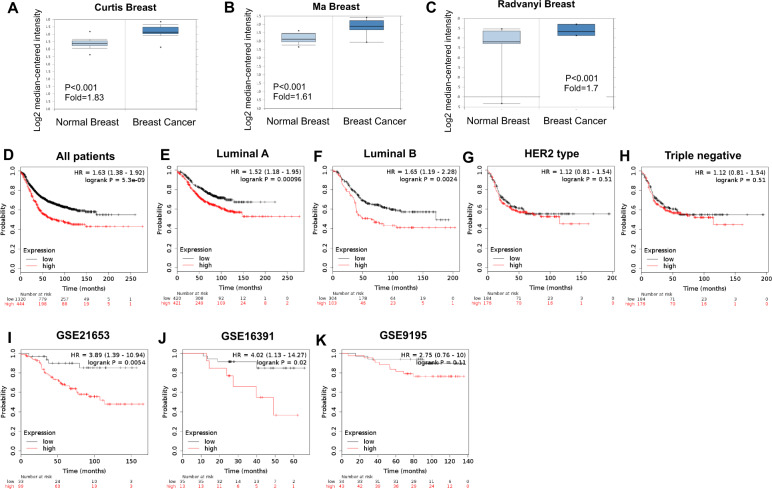
RNF181 is elevated in breast cancer and correlates with poor survival in endocrine therapy patients. **a**–**c** RNF181 is elevated in breast cancer compared with normal breast tissue in multiple clinical cohorts (https://www.oncomine.org). **d** RNF181 expression is correlated with poor survival in breast cancer patients (https://kmplot.com). **e** RNF181 expression is correlated with poor survival in Luminal A type breast cancer patients (https://kmplot.com). **f** RNF181 expression is correlated with poor survival in Luminal B type breast cancer patients (https://kmplot.com). **g** There is no statistical correlation between RNF18 and survival in HER2 type breast cancer patients (https://kmplot.com). **h** There is no statistical correlation between RNF181 and survival in HER2 type breast cancer patients (https://kmplot.com). **i**–**k** RNF181 correlates with poor endocrine therapy outcome in multiple clinical cohorts (https://kmplot.com).

We first investigate the possible role of RNF181 from public available database. From the ONCOMINE database, we observe that RNF181 is elevated in breast cancer compared with normal breast tissue in multiple clinical cohorts (https://www.oncomine.org/resource/login.html) (Fig. 1a–c). The survival data analysis shows that RNF181 is correlated with shorter progression-free survival in all breast cancer patients (Fig. 1d). In the subtype analysis, RNF181 correlates with poor survival in Luminal A type and Luminal B type breast cancer, but not with HER2 type and triple negative breast cancer types (Fig. 1e–h). We further analyze the prognostic effect of RNF181 in endocrine-treated patients. Three independent clinical cohorts show that RNF181 correlates with poor survival in breast cancer patients with endocrine therapy (Fig. 1i–k).

### **RNF181 depletion inhibits breast cancer progression in vivo and in vitro**

**Fig. 2:**
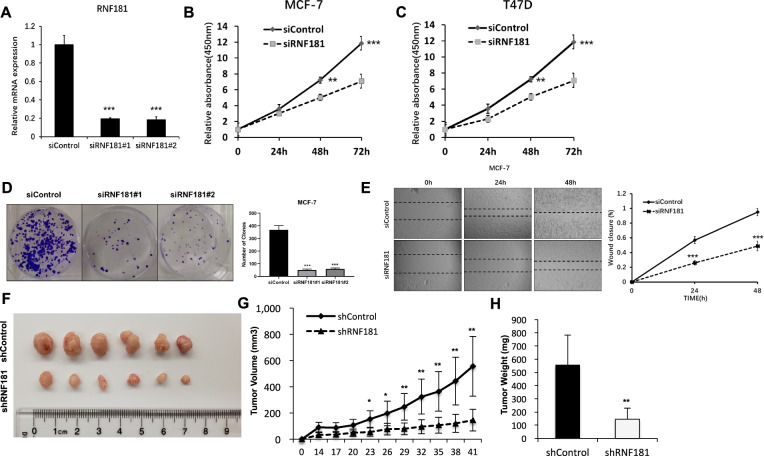
RNF181 depletion inhibits breast cancer progression in vivo and in vitro. **a** RNF181 knockdown efficiency in breast cancer cell. MCF-7 cells was transfected with siControl or siRNF181. The RNF181 mRNA levels were measured by Q-PCR. *P < 0.05; **P < 0.01; ***P < 0.001 for target gene expression comparison. **b** and **c** Depletion of RNF181 inhibits the proliferation of breast cancer cells. MCF-7 and T47D cells were transfected with siControl or siRNF181. After 24 h, the assay was CCK8 used to determine the cellar metabolic activity at indicated time points after infection. Experiments were done in triplicates. *P < 0.05; **P < 0.01; ***P < 0.001 for cell growth comparison. **d** Clone formation assay of MCF-7 cells transfected with indicated 50 nM RNF181 siRNA (mix of #1 and #2) or 50 nM control siRNA. Quantification of clone formation is shown at the indicated time points. Data are presented as ±SD. **P < 0.01, ***P < 0.001 (student’s *t* test). **e** Wound-healing assay of MCF-7 cells were transfected with siControl or siRNF181. Quantification of wound closure at the indicated time points. Data are presented as ±SD. **P < 0.01, ***P < 0.001. **f**–**h** MCF-7 cells were stably transfected with lentivirus carrying scrambe shRNA or RNF181 shRNA. Female NOD scid gamma (NSG) mice were estrogen-supplemented by implantation of slow-release 17β-estradiol pellets (0.72 mg/90-d release; Innovative Research of America) one day before MCF-7 tumor cell injection into the mammary fat pad (2 × 10^6^ MCF-7 cells suspended in 100 ul Matrigel solution). MCF-7 tumor xenografts were measured every 3 days and the tumor volume were calculated by length × width^2^ /2. The mice were sacrificed at 6 weeks after transplant. The tumor growth curve, tumor weight, and photograph were shown in figure **f**, **g** and **h** respectively.

In order to investigate the impact of RNF181 on breast cancer phenotypes, we deplete RNF181 in breast cancer cells (Fig. 2a). WST assay shows that RNF181 depletion significantly decreases breast cancer cell proliferation in MCF-7 and T47D cells (Fig. 2b, c). Clone formation assay shows that RNF181 depletion dramatically inhibits the clone formation capacity in MCF-7 cells (Fig. 2d). Beside, the wound-healing assay shows that RNF181 knockdown decreases the wound closure speed in MCF-7 cells (Fig. 2e). Then we further investigate the role of RNF181 in vivo by xenograft mice model. Our data show that RNF181 depletion inhibits the tumor growth speed in vivo (Fig. 2f–h).

### **RNF181 correlates with ERα protein level in human samples and controls ERα target gene expression in whole genomic scale**

**Fig. 3:**
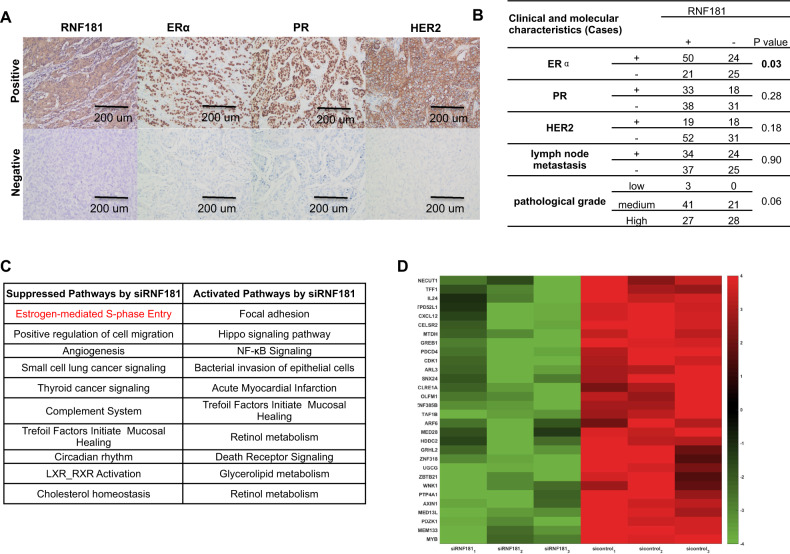
RNF181 correlates with ERα protein level in human samples and controls ERα target gene expression in whole genomic scale. **a** Examples of positive/negative RNF181, ERα, PR, and HER2 staining in breast tumor samples were shown by ×100 magnification. **b** RNF181 expression correlates with ERα level in human breast cancer samples. The correlation analysis between RNF181 expression and molecular/clinical characteristics in breast cancer samples was shown. *P* value < 0.05 was considered as statistical significance. **c** Top 10 signaling pathways significantly decreased/increased by RNF181 depletion in MCF7 cells. The pathway-enrichment analysis was used by the threshold *P* < 0.001 and fold change > 2 to derive regulated genes. RNF181 was depleted by siRNA (mix of siRNF181 #1 and siRNF181 #2) or treated with siControl. After 48 h, the whole mRNA was extracted for RNA sequence analysis. The siControl and siRNF181 were done in triplicates. **d** The heat-map graph shows the ERα regulating genes, which is significantly inhibited by RNF181 depletion in MCF-7 cells.

In order to investigate the correlation between RNF181 expression and breast cancer molecular biomarkers, 120 breast cancer tissues are collected for immunohistochemistry (IHC) analysis. We examine the protein expression levels including RNF181, ERα, PR, and HER2 (Fig. 3a). Besides, the pathological grade and lymph node metastasis data are also collected. The IHC analysis implicate that RNF181 expression correlates with ERα protein level, but no correlation with other with other molecular and clinical characteristics (Fig. 3b). In order to approach the function of RNF181 in breast cancer cells in an unbiased way, we deplete RNF181 in MCF-7 cells for the whole genomic expression analysis. The bioinformatics analysis shows that RNF181 depletion inhibits several signaling, including ERα signaling and angiogenesis signaling, while activates several pathways, such as NFKB signaling and cell death signaling (Fig. 3c). Since ERα signaling is pre-dominant in ERα positive breast cancer cells, we further investigates the change of ERα target gene in whole genomic scale. The heat-map shows RNF181 depletion significantly decrease a group of ERα genes, including PS2 and GREB1 (Fig. 3d).

### **RNF181 facilitates ERα signaling in breast cancer cells**

**Fig. 4:**
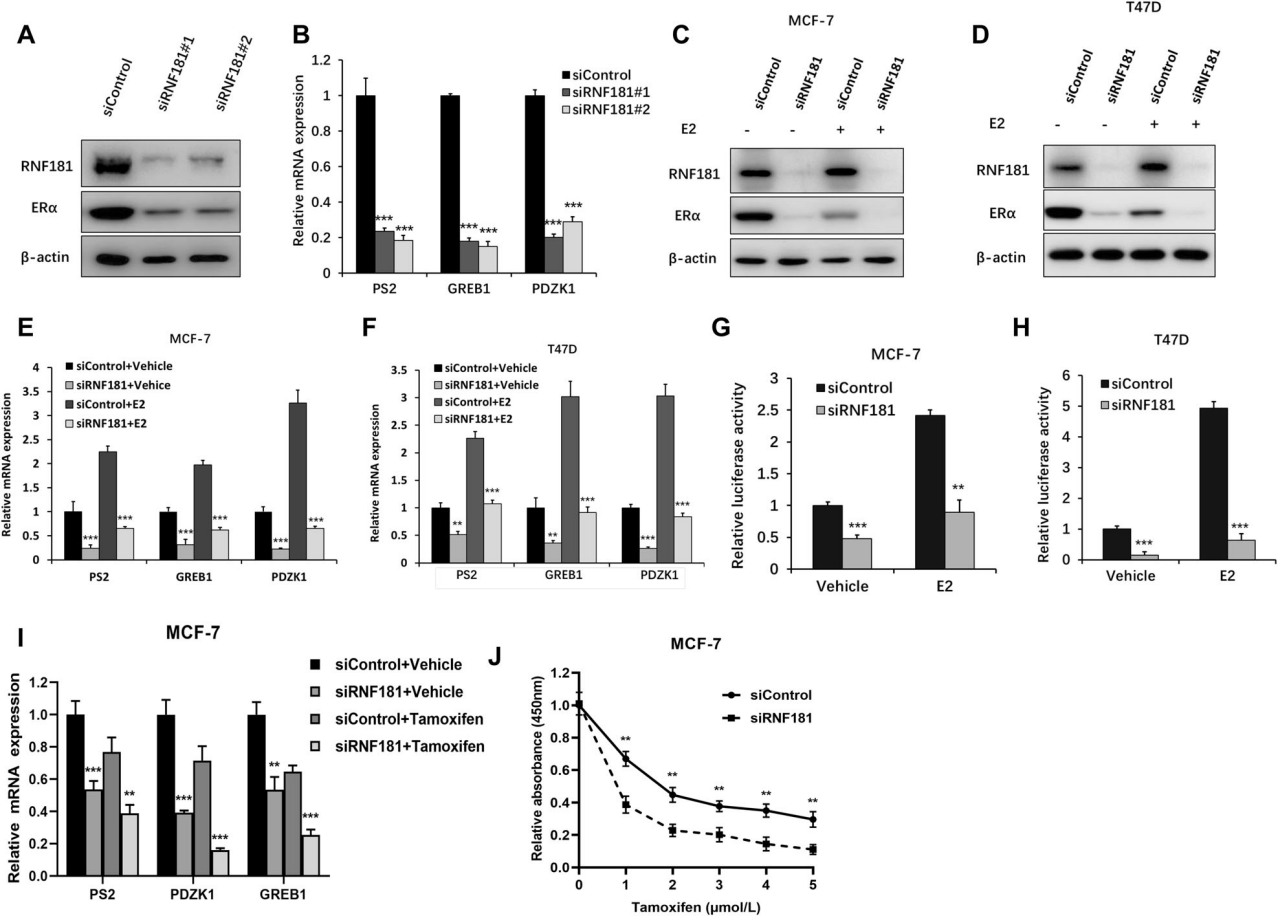
RNF181 facilitates ERα signaling in breast cancer cells. **a** RNF181 consumption decreased ERα protein levels in MCF-7 cells. MCF-7 cells were transfected with siControl or siRNF181. After 48 h, cells were harvested for western blot analysis. RNF181 and ERα protein levels were determined by Western blot. Actin was used as internal control. **b** RNF181 consumption decreased ERα target gene expression in MCF-7 cells. MCF-7 cells were transfected with siControl or siRNF181. After 48 h, total RNA was extracted for gene expression analysis. *P < 0.05; **P < 0.01; ***P < 0.001 for target gene expression comparison. **c** RNF181 depletion decreases ERα protein levels in both vehicle and E2-treated conditions in MCF-7 cells. MCF-7 cells were transfected with siRNF181 or siControl. After 48 h, cells were treated with either ethanol or 10 nM estradiol for 6 h. RNF181 and ERα protein levels were determined by Western blot analysis. Actin was used as internal control. **d** RNF181 depletion decreases ERα protein levels in both vehicle and E2-treated conditions in T47D cells. T47D cells were transfected with siRNF181 or siControl. After 48 h, cells were treated with either ethanol or 10 nM estradiol for 6 h. RNF181 and ERα protein levels were determined by Western blot analysis. Actin was used as internal control. **e** RNF181 depletion decreases ERα target genes in both vehicle and E2-treated conditions in MCF-7 cells. MCF-7 cells were transfected with siRNF181 or siControl. After 48 h, cells were treated with either ethanol or 10 nM estradiol for 6 h. Total RNA was prepared and the expression of the endogenous ER alpha target genes, PS2, GREB1, and PDZK1 were determined by qPCR. Shown are the results from three experiments. *P < 0.05; **P < 0.01; ***P < 0.001 for target gene expression comparison. **f** RNF181 depletion decreases ERα target genes in both vehicle and E2-treated conditions in T47D cells. T47D cells were transfected with siRNF181 or siControl. After 48 h, cells were treated with either ethanol or 10 nM estradiol for 6 h. Total RNA was prepared and the expression of the endogenous ER alpha target genes, PS2, GREB1, and PDZK1 were determined by qPCR. Shown are the results from three experiments. *P < 0.05; **P < 0.01; ***P < 0.001 for target gene expression comparison. **g** RNF181 depletion affects ERE-luciferase activity in MCF-7 cells. MCF-7 cells were transfected with siRNF181 or siControl together with ERE luciferase reporter plasmid. Cells were treated with 10 nM estradiol or vehicle. Luciferase activity was measured 48 h after transfection. Shown are the results from three experiments. *P < 0.05; **P < 0.01; ***P < 0.001 for luciferase activity comparison. **h** RNF181 depletion affects ERE-luciferase activity in T47D cells. T47D cells were transfected with siRNF181 or siControl together with ERE luciferase reporter plasmid. Cells were treated with 10 nM estradiol or vehicle. Luciferase activity was measured 48 h after transfection. Shown are the results from three experiments. *P < 0.05; **P < 0.01; ***P < 0.001 for luciferase activity comparison. **i** RNF181 depletion decreases ERα target genes in both vehicle and tamoxifen-treated conditions in MCF-7 cells. MCF-7 cells were transfected with siRNF181 or siControl. After 48 h, cells were treated with either ethanol or 0.5 uM tamoxifen for 6 h. Total RNA was prepared and the expression of the endogenous ER alpha target genes, PS2, GREB1, and PDZK1 were determined by qPCR. Shown are the results from three experiments. *P < 0.05; **P < 0.01; ***P < 0.001 for target gene expression comparison. **j** RNF181 depletion sensitizes tamoxifen inhibition effect in MCF-7 breast cancer cells. MCF-7 cells were transfected with siRNF181 or siControl. After 48 h, cells were plated into 96-well plate, while each well contained 4000 cells. The indicated tamoxifen concentrations were used for 48 h. The numbers of the cells were determined via CCK8 kit for the cellar metabolic activity. Experiments were done in triplicates. *P < 0.05; **P < 0.01; ***P < 0.001 for cell growth comparison.

We utilize two independent siRNAs to carry out the experiments. The immuno-bloting shows that RNF181 depletion significantly decreases ERα proein level (Fig. 4a). The QPCR assay shows that RNF181 depletion decreases the expression of ERα target genes, including GREB1 and PS2 (Fig. 4b). We further test RNF181 effect on ERαsignaling in both vehicle and E2-treated conditions. RNF181 depletion could decrease ERα protein level in vehicle and E2-treated conditions in both MCF-7 and T47D cells (Fig. 4c, d). Consistently, RNF181 depletion could dramatically decrease ERα target gene expression in MCF-7 and T47D cells, including PS2, GREB1, and PDZK1 (Fig. 4e, f). In order to determine if RNF181 knockdown could affect ERα transcriptional activity, we measure estrogen response element (ERE) luciferase activity in both MCF-7 and T47D cells. The luciferase assay shows that RNF181 depletion decreases ERE luciferase activity in both MCF-7 and T47D cells (Fig. 4g, h). We further investigated the role of RNF181 in ER signaling under tamoxifen-treated conditions. RNF181 depletion could decrease ERα target gene expression in MCF-7 under anti-estrogen condition, including GREB and PS2 (Fig. 4i). Besides, the cell viability assay showed that RNF181 depletion could sensitize tamoxifen inhibition effect in MCF-7 cells (Fig. 4h). We further utilized siRNA, which targeted the UTR regions of RNF181. The data showed that three independent siRNAs could effectively inhibited ERα protein level, ERα target genes expression, and ERE luciferase activity in MCF-7 cells (Supplementary fig. 1A–E). Further rescue experiments showed that RNF181 overexpression could rescue MCF-7 cell proliferation, migration and clone formation, which were inhibited by RNF181 UTR siRNAs (Supplementary fig. 1F–H). Besides, we also validated the effect of RNF181 via siRNAs provided from Depmap database. The data also showed that RNF181 depletion inhibited ERα signaling, breast cancer cell proliferation, migration, and clone formation in MCF-7 cells (Supplementary fig. 2).

### **RNF181 associates with ERα and modulates ERα stability**

**Fig. 5:**
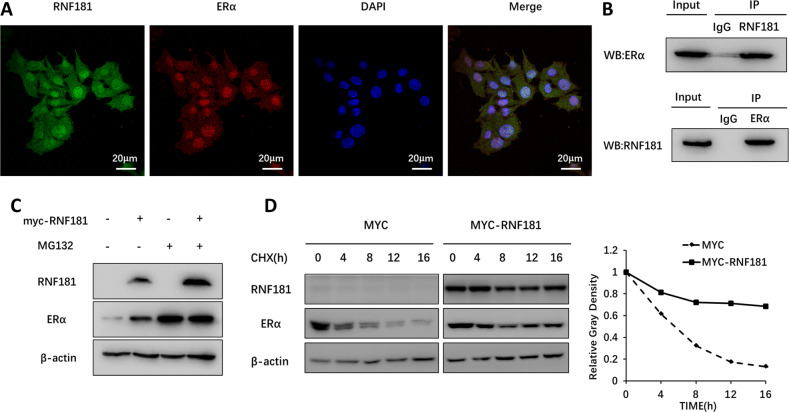
RNF181 associates with ERα and modulates ERα stability. **a** Intracellular localization analysis of RNF181 and ERα by immunofluorescence assay. MCF7 cells were cultured in normal medium before fixation. Intracellular localization of RNF181 (green) and ERα (red) were shown. Nuclei (blue) were stained with 4',6-diamidino-2-phenylindole (DAPI). **b** Co-IP assay reveals association between endogenous RNF181 and ERαin MCF7 cells. MCF-7 cells were harvested with RIPA lysis buffer. CO-IP was performed using antibody as indicated. **c** In the presence of the proteasome inhibitor MG132, the stabilization effect of RNF181 on ERα did not further increase ERα protein levels. HEK293 cells were transfected with 2 µg RNF181 plasmid and 0.5 µg Myc-tag or Myc-RNF181 plasmids. After 24 h, cells were treated with 10 uM MG132/vehicle for 6 h. Cell lysates were prepared for Western blot analysis. The results are representative for three independent experiments. **d** RNF181 increases ERα half-life in HEK293 cells. HEK293 cells were transfected with HA-ERα plasmid and Myc-tag or Myc-ZNF213 plasmids. After 24 h, cells were treated with 100 µM cycloheximide/vehicle for indicated times. Cell lysates were prepared for Western blot analysis. The results are representative for three independent experiments. The ERα relative density was measured by Image J software.

We further investigate the localization of RNF181 and ERα in breast cancer cells. The immuno-staining shows that ERα is mainly located in the nuclear, while RNF181 locates both in the cytosol and nuclear (Fig. 5a). The endogenous immuno-precipitation shows that RNF181 could interact with ERα in MCF-7 cells (Fig. 5b). Since RNF181 could associate with ERα in breast cancer cells, we further investigate the biological effect of such interaction. Since ERα could regulate its own expression, making it difficult to distinguish direct effects of RNF181 on ERα mRNA or protein levels in the cell line. We utilize HEKC293 cells to investigate the mechanism. Co-transfection of ERα and RNF181 in HEK293 cells shows that RNF181 could increase ERα protein level, which effect could be minimized with the presence of the proteasome inhibitor MG132 (Fig. 5c). The protein half-life assay shows that RNF181 could increase the protein stability of ERα (Fig. 5d).

### **RNF181 associates with AF1 domain of ERα through its RING domain**

**Fig. 6:**
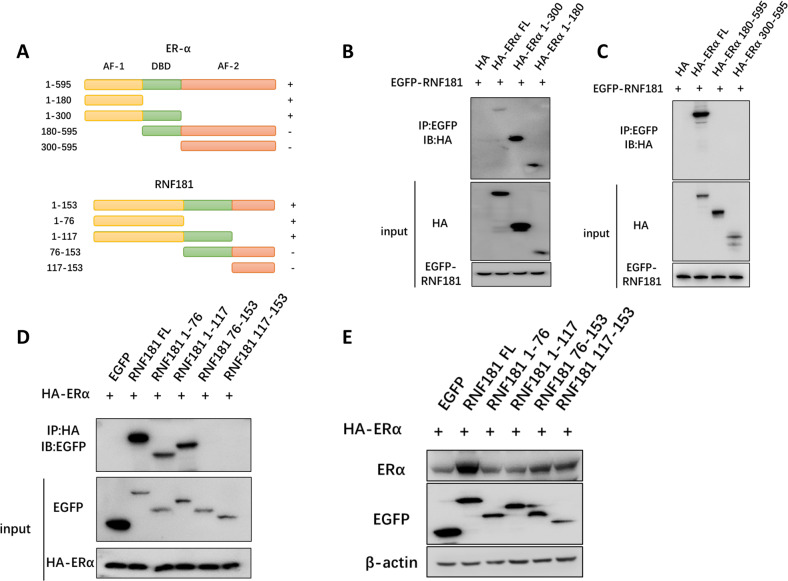
RNF181 associates with AF1 domain of ERα through its RING domain. **a** ERα domain structure and deletion mutants were used in the study and RNF181 full length and deletion mutants were used in the study. **b** and **c** ERα interacts with RNF181 through its AF1 domain. HEK293 cells were transfected with 2 µg Myc-RNF181 together with HA- ERα full length or mutants (ΔAF1, ΔAF1 + ΔDBD, ΔAF2 and ΔAF2 + ΔDBD). After 24 h, cells were treated with 10 uM MG132 for 6 h. Then the cells were harvested with NP-40 lysis buffer. CO-IP was performed using Myc antibody. The possible interacted ERα domains were detected by HA antibody. **d** RING domain is required for RNF181 to interaction with ERα. HEK293 cells were transfected with 2 µg HA- ERα together with GFP-RNF181 full length or mutants (ΔN-terminal, ΔRING + ΔN-terminal, ΔC-terminal, ΔRING + ΔC-terminal). After 24 h, cells were treated with 10 uM MG132 for 6 h. Then the cells were harvested with NP-40 lysis buffer. CO-IP was performed using HA antibody. The possible interacted RNF181 domains were detected by GFP antibody. **e** The intact RNF181 protein is require for its stabilization effect on ERα. HEK293 cells were transfected with 2 µg Flag- ERα and 0.5 µg GFP-RNF181 full length or mutants (ΔN-terminal, ΔRING + ΔN-terminal, ΔC-terminal, ΔRING + ΔC-terminal). The ERα protein levels were detected via western blotting analysis.

We further characterize the interaction domains between RNF181 and ERα. ERα is composed of three functional domains: AF1 domain, DNA-binding domain, and AF2 domain, while RNF181 contains one functional RING domain (76aa-117aa). We make the ERα variants (AF1 domain, AF1 + DBD domains, AF2 domain and AF2 + DBD domains) and RNF181 variants (N-terminal: 1-76 aa; RING domain: 76–117 aa; C-terminal: 117-153 aa) (Fig. 6a). Immuno-precipitation implicates that the AF1 domain of ERα is required for its interaction with RNF181 (Fig. 6b, c), while the RING domain of RNF181 is required for its interaction with ERα (Fig. 6d). However, co-transfection of ERα with RNF181 variants shows that only the full length of RNF181 could exert its stabilization function on ERα protein (Fig. 6e).

### **RNF181 facilitates K63-linked poly-ubiquitination of ERα**

**Fig. 7:**
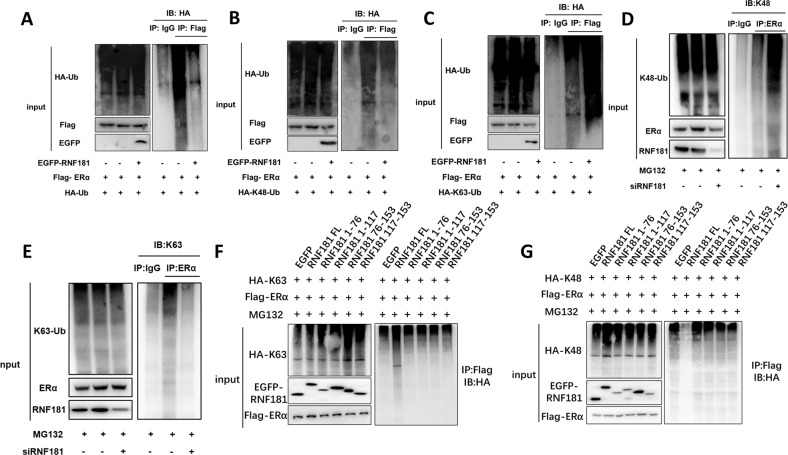
RNF181 facilitates K63-linked poly-ubiquitination of ERα. **a** RNF181 decreases poly-ubiquitination of ERα. HEK293 cells were transfected with 2 µg ERα plasmid, 0.5 µg HA Ub plasmid and 0.5 µg Myc-tag or Myc-RNF181 plasmids. The cell extracts were immunoprecipitated with HA antibody. The poly-ubiquitinated ERα was detected via western blotting analysis. **b** RNF181 decreases K48-linked poly-ubiquitination of ERα. HEK293 cells were transfected with 2 µg ERα plasmid, 0.5 µg HA-K48 Ubi plasmid and 0.5 µg Myc-tag or Myc-RNF181 plasmids. The cell extracts were immunoprecipitated with HA antibody. The K48 specific poly-ubiquitinated ERα was detected via western blotting analysis. **c** RNF181 promotes K63-linked poly-ubiquitination of ERα. HEK293 cells were transfected with 2 µg ERα plasmid, 0.5 µg HA-K63 Ubi plasmid and 0.5 µg Myc-tag or Myc-RNF181 plasmids. The cell extracts were immunoprecipitated with HA antibody. The K63 specific poly-ubiquitinated ERα was detected via western blotting analysis. **d** RNF181 depletion increases endogenous K48-linked poly-ubiquitination of ERα in breast cancer cells. MCF-7 cells were transfected with 50 uM siRNF181 siRNA or siControl. After 48 h, cells were treated with MG132 for 6 h. The cell extracts were immunoprecipitated with ERα antibody (D8H8, Cell signaling Technology). The K48 specific poly-ubiquitinated ERα was detected via K48 specific Ub antibody (A101, R&D company). **e** RNF181 depletion inhibited endogenous K63-linked poly-ubiquitination of ERα in breast cancer cells. MCF-7 cells were transfected with 50 uM siRNF181 siRNA or siControl. After 48 h, cells were treated with MG132 for 6 h. The cell extracts were immunoprecipitated with ERα antibody (D8H8, Cell signaling Technology). The K63 specific poly-ubiquitinated ERα was detected via K63 specific Ub antibody (HWA404, Enzo life science). **f** The intact RNF181 protein is require for the inhibition of K48-linked poly-ubiquitination on ERα. HEK293 cells were transfected with 2 µg ERα plasmid, 0.5 µg HA-K48 Ubi plasmid and 0.5 µg GFP-RNF181 full length or mutants (ΔN-terminal, ΔRING + ΔN-terminal, ΔC-terminal, ΔRING + ΔC-terminal) plasmids. The cell extracts were immunoprecipitated with HA antibody. The K48 specific poly-ubiquitinated ERα was detected via western blotting analysis. **g** The intact RNF181 protein is require for the induction of K63-linked poly-ubiquitination on ERα. HEK293 cells were transfected with 2 µg ERα plasmid, 0.5 µg HA-K63 Ubi plasmid and 0.5 µg GFP-RNF181 full length or mutants (ΔN-terminal, ΔRING + ΔN-terminal, ΔC-terminal, ΔRING + ΔC-terminal) plasmids. The cell extracts were immunoprecipitated with HA antibody. The K63 specific poly-ubiquitinated ERα was detected via western blotting analysis.

Since RNF181 is a putative E3 ubiquitin ligase, we further investigate the role of RNF181 on ERα ubiquitination. The ubiquitination-based immuno-precipitation shows that RNF181 could inhibit ERα overall poly-ubiquitination (Fig. 7a). Since K48-linked ubiquitination is the most common degradation manner, we examine the RNF181 effect on K48-linked ubiquitination of ERα, which implicates that RNF181 could inhibit K48-linked ubiquitination of ERα (Fig. 7b). However, RNF181 could significantly increase K63-linked poly-ubiquitination on ERα protein (Fig. 7c). We further investigated ERα poly-ubiquitination in MCF-7 cells. Endogenous ERα pull down assay together with immuno-bloting for K48 and K63-linked ubiquitin signals showed that RNF181 depletion could significantly increase K48-linked ubiquitination of endogenous ERα, while decrease K63-linked ubiquitination of endogenous ERα in MCF-7 cells (Fig. 7d, e). In order to decide which is the functional domain for RNF181 to modulate ERα ubiquitination, RNF181 full length or deletion constructs (N-terminal: 1-76 aa; RING domain: 76–117 aa; C-terminal: 117-153 aa) together with ERα are transfected into HEK293 cells. Interestingly, only full length of RNF181 could inhibit K48-linked ERα ubiquitination and promote K63-linked ERα ubiquitination (Fig. 7f, g). This indicates all the domains of RNF181 is involved in modulate ERα ubiquitination.

## Discussion

**Fig. 8:**
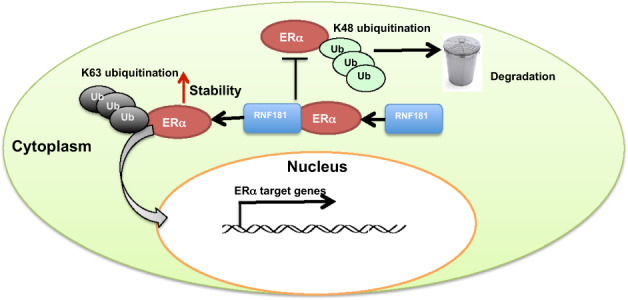
The hypothesis model of RNF181 regulating ERα signaling in breast cancer. RNF181 interacts with ERα protein, stabilizes ERα and facilitates its signaling activation and breast cancer progression.

Our study reports that the E3 ubiquitin ligase RNF181 interacts with and stabilizes ERα protein, possibly through K63-linked ubiquitination, which subsequently facilitates ERα signaling and breast cancer cell progression (Fig. 8). Interestingly, RNF181 could associate with ERα protein level in human breast cancer samples and correlates with poor survival in endocrine therapy patients. Our study provides a novel non-genomic regulation of ER stability control. Based on these data, we can propose that the selective modulators or inhibitors, which control RNF181 activity or expression could a promising strategy for ERα positive breast cancer therapeutics.

The importance of ERα signaling in the carcinogenesis of breast tumor has been identified for more than 30 years, since 70% of the breast cancers are ERα positive. The risk of breast cancer is also correlated with ERα expression level in breast tissue [19]. High level of ERα expression in breast cancer leads to enhance ERα signaling activity and cell proliferation [20]. Based on the importance of ERα, it is a suitable target for breast cancer therapy. The selective estrogen receptor modulators, including tamoxifen, are standard therapy for ERα positive patients. However, the occurrence of endocrine resistance is a headache problem for breast cancer therapy [21]. Interestingly, most of the endocrine resistant breast tumors still maintain the expression of ERα protein, which might implicates the participation of ERα in mediating endocrine resistance [22]. Thus, modulating of ERα expression and stability could be a promising strategy for breast cancer therapeutics and endocrine resistance.

Among hundreds of the E3 ubiquitin ligases, the members of the RING proteins attracted the attentions because of the uncommon ubiquitination members and the regulatory functions [23]. Unlike the classical E3 ligases, a proportion of RING family E3 ligases seem to preferentially catalyze atypical ubiquitinations, which does not link to protein degradation and turnover [24]. For example, RNF31 coupled with RBCK1 promote the linear ubiquitination of IKKr and facilitate NFKB signaling trans-activation [25, 26]. Recently studies reveal that several RING family members participate in ERα signaling activation and breast cancer progression via non-degradation related ubiquitinations [27, 28]. For example, RNF31 could associate with ERα, mono-ubiquitinate ERα and promote breast cancer proliferation [18]. Our previous studies identified a few E3 ubiquitin ligases in modulating ERα signaling via either genomic regulations or post-translational modifications, including TRIM56 and SMURF1 [27, 28]. In our current study, we uncover a novel regulatory mechanism between RNF181 and ERα, which provides both novel knowledge of RNF family members in ERα function and a promising strategy for ERα positive cancer therapeutics.

RNF181 belongs to the RING E3 ubiquitin ligase family, while the RING domain functions to catalyze the ubiquitin ligase reaction. RNF181 is critical for a few physiological functions [29]. For example, RNF181 functions to modulate integrin to facilitate platelet aggregation [30]. Interestingly, RNF181 is elevated in quite a few human cancers. RNF181 could interact with CARD11 and promote NKFB pathway in lymphoma [31]. Besides, RNF181 is also proved to facilitate colon cancer survival and angiogenesis [32]. Here, our data implicate that RNF181 is increased in human breast cancer and relates to poor outcome in endocrine therapy patients. RNF181 could promote breast cancer progression in vivo and in vitro and facilitate ERα signaling. Our study proves that RNF181 stabilizes ERα protein via a non-proteolytic ubiquitination manner, which provide a novel insight of the RING family protein in ERα signaling and breast cancer progression.

In conclusion, we identified an interesting E3 ligase RNF181 in facilitating ERα signaling in breast cancer cells. RNF181 could promote breast cancer cell invasion and proliferation via stabilizing ERα protein. As a novel modulator of ERα signaling, disturbing RNF181 activity or affecting RNF181 expression could be a plausible way to treat luminal types of breast cancer.

## Materials and methods

### Cell culture

MCF-7, T47D, and HEK293 cells are got from American Type Culture Collection (ATCC). T47D cells are cultured with RPMI-1640 (42401, Life Technologies) supplemented with 2 mM L-glutamine (25030, Life Technologies) and 10% FBS. MCF-7 and HEK293 are culture with Dulbecco’s Modified Eagle’s Medium that contains 4,5 g/L glucose and 4 mM L-glutamine (DMEM, 41965, Life Technologies) supplemented with 10% Fetal Bovine Serum (FBS, 10270, Life Technologies). All cell lines are characterized by cell line authentication. The cell line authentication via Short Tandem Repeat (STR) is performed via PowerPlex 21 system. The STR data of MCF-7 and T47D cell lines are found consistent with STR data in ATCC.

### RNA extraction and qPCR analysis

Total RNA was used to extract by RNeasy plus mini kits (Tiangen). Real-time PCR was showed as previously described [33]. 36B4 was used for internal reference. The primer sequences were displayed here. RNF181: F: cac aga cga gat aag gct cga a; R: tgg cca ggt ctg tga tca at. 36B4: F: ggc gac ctg gaa gtc caa ct; R: cca tca gca cca cag cct tc. GREB1 F: CGT GTG GTG ACT GGA GTA GC, R: ACC TCT TCA AAG CGT GTC GT. ER F: GCT ACG AAG TGG GAA TGA TGA AAG, R: TCT GGC GCT TGT GTT TCA AC. PS2 (TFF1) F: TGG GCT TCA TGA GCT CCT TC, R: TTC ATA GTG AGA GAT GGC CGG. The siRNA, shRNA and CRISPR g-RNA sequences were provided in supplementary table 1.

### Quantification of cell viability

MCF-7 and T47D cells were transfected with siRNF181 or siControl in 24-well plates. Twenty-four hours after transfection, the cells number was countered and 4000 cells were seeded into 96-well plates. The relative cell viability was measured at indicated time points. Cell numbers were determined using the WST-1 cell proliferation reagent as previously described [34].

### Wound healing assay

Fifty nM RNF181 siRNA or siControl were transfected into MCF-7 cells. After twenty-four hours, cells were seeded into 12-well paltes with 1% FBS. The cells were 100% confluence. The yellow pipette tips were applied for straight scratch. The wound distance was measured at indicated time points and normalized with starting time point. The wound healing recovery was expressed as: [1-(Width of the wound at a given time/width of the wound at *t* = 0)] × 100%

### Clone formation assay

MCF-7 were seeded in six-well plates overnight and treated with 50 nM RNF181 siRNA or 50 nM siControl. Twenty-four hours post-transfection, the cells were washed with PBS, trypsinized and plated at low density (5000 cell/well in six-well plate). The cells were cultured for 10 days and the medium was refreshed every 2 days. The colonies were stained with crystal violet. The number of the clones in a given area was counted for each condition.

### Xenograft tumor model

MCF-7 cells were infected with shControl virus or shRNF181 virus. After 48 h of infection, cells were selected with 1 ug/ml puromycin for 3 days. The female nonobese diabetic-SCID mice were implanted with slow-relase 17 beta-estradiol pellets (0.72 mg/90-day, Innovative Research of America). After 24 h, about one million MCF-7 cells together with matrigel solution were injected into the mammary fat pad for each mouse. The tumor sizes were measured every 3 days. After 6 weeks, the mice were sacrificed and the tumors were weighted and photographed. The experiments were performed under the protocols approved by ethnic committee of Xinxiang Medical University.

### Western blotting

Cells were harvested and lysed with RIPA buffer. Proteins were separated by electrophoresis on SDS-polyacrylamide gel electrophoresis (PAGE) and electro-transferred to PVDF membrane. The antibodies used in this study were listed here: Anti- ERα (D8H8, 8644, Cell signaling Technology); Anti- ERα (SC-56833, Santa Cruz); Anti-RNF181 (SAB1401685, Sigma); Anti-HA (MMS-101R, COVANCE); Anti-myc (9E10, ab32, Abcam); Anti-myc (Ab9106, Abcam); RBCK1 (Ab108479, Abcam); Anti-Flag (Ab49763, Abcam); Anti-GFP (Ab290, Abcam). Membranes were then washed with PBS for three times and incubated with secondary antibodies Peroxidase-Conjugated AffiniPure Goat Anti-Mouse IgG or Goat Anti-Rabbit IgG. Fluorescent signals were visualized with ECL system (amersham imager 600, USA).

### Luciferase assay

The luciferase activity of estrogen signaling activity was performed using the Dual-Luciferase Reporter kit (Promega, Germany). The ERE luciferase reporter was transfected together with the Renilla plasmid into the cells. Luciferase activity was measured after 24 h.

### Co-immunoprecipitation assay

Immunoprecipitation was performed as described in previous study [35]. The MCF-7 total cell lysls were pre-cleared with rabbit IgG for 2 h and subsequently immunoprecipitated with ERα antibody (SC8005, Santa Cruz) overnight, while rabbit IgG (Santa Cruz) was used as the negative control. The bounded protein was analyzed by Anti-RNF181 (SAB1401685, Sigma). For the overexpression experiment, HEK293 cells were transfected with 5 ug GFP-RNF181 (Full length or deletion domains) and ERα plasmid (Full length or deletion domains) in 10 cm dish. Cell lysates were pre-cleared with IgG and subsequently incubate with GFP (Ab290, Abcam) antibody, while rabbit IgG was used as the negative control. The bound proteins were analyzed by western blotting.

### Poly-ubiquitination detection assay

To directly detect the enriched overall ubiquitinated, K48-linked or K63-linked ubiqutinated ERα from the cell extracts, HEK293 cells were transfected with 4 ug Ub, 4 ug K48 Ubi or 4 ug K63 Ubi plasmid, 2 ug ERα together with 0.5 ug Flag-RBCK1 or Flag-vector. After 48 h, cells were treated with 10 uM MG132 and then the total protein was extracted and pre-cleared with 20 ul protein A (santa cruz, SC-2001) for 2 h. The supernatant was collected and immunoprecipitated by ERα antibody. Western blot with HA antibody was performed to detect K48 and K63 poly-ubiquitinated ERα.

### Immunofluorescence assay

MCF-7 cells were fixed with 4% paraformaldehyde in PBS for 10 min, permeabilized with 0.2% Triton X-100 for 5 min, and blocked by 5% BSA in PBS for 1 h. A rabbit Anti-RNF181 (SAB1401685, Sigma) rabbit antibody and mouse anti-ERα monoclonal antibody (SC-56833) were used, followed by Alexa Flour 647 (Invitrogen) anti-rabbit antibody and FITC-conjugated anti-mouse antibodies (Jackson ImmunoResearch, West Grove, PA). As negative controls, the samples were incubated with the secondary antibodies without primary antibodies. Images were acquired under conditions fulfilling the Nyquist criterion using Nikon A + laser scanning confocal system with a 60X oil NA1.4 objective and pinhole size of 1.0 Airy Unit. The acquired pictures were further processed and assembled using ImageJ.

### Clinical breast tumor samples

One hundred and twenty formalin-fixed paraffin-embedded breast cancer samples were collected from the Department of Pathology, Shandong Qilu Hospital. All the breast tumors samples were examined by ERα status, PR status, HER2 status by pathological specialists. The pathological grade plus lymph node metastasis status of each sample was also examined by pathological specialists. This study was reviewed and approved by the Ethical Board at the Qilu Hospital of Shandong University with written informed consent from all the patients.

### RNA sequence analysis

The global gene expression analysis (siControl and siRNF181) was based on RNA sequencing platform from BGI (Beijing Genomic Institute). The RNA sequence data are deposited in the Gene Expression Omnibus database (Assessing number: GSE143947). Analysis was performed for differentially expressed genes (*P* < 0.01 and fold change > 2) by Ingenuity Pathway Analysis (IPA).

### Statistics

Student’s *t* test, Pearson correlation coefficient, and Cox regression analysis were used for comparisons. A *P* value of < 0.05 was considered to be significant.

## Supplementary information

Supplementary Figure legends

Supplementary figures

Supplementary tables
