# Supplementary material for: The ubiquitin ligase RNF181 stabilizes ERα and modulates breast cancer progression
Source: Oncogene. 2020 Sep 24;39(44):6776–88. doi: 10.1038/s41388-020-01464-z (PMC7605433; doi:10.1038/s41388-020-01464-z)
Supplement: Supplementary file 1 — Supplementary Figure legends [file 41388_2020_1464_MOESM1_ESM.docx]

**Supplementary Figure 1**

**A:** RNF181 depletion via UTR siRNAs could inhibit ERα protein levels in MCF-7 cells.

**B:** RNF181 depletion via UTR siRNAs could effective inhibit RNF181 mRNA level in MCF-7 cells.

**C:** RNF181 depletion inhibited ERα protein level, which effect could be further rescued by RNF181 overexpression in MCF-7 cells.

**D:** RNF181 depletion inhibited ERα target gene expression, which effect could be further rescued by RNF181 overexpression in MCF-7 cells.

**E:** RNF181 depletion inhibited ERE luciferase activity, which effect could be further rescued by RNF181 overexpression in MCF-7 cells.

**F:** RNF181 depletion inhibited breast cancer cell proliferation, which effect could be further rescued by RNF181 overexpression in MCF-7 cells.

**G:** RNF181 depletion inhibited clone formation capacity, which effect could be further rescued by RNF181 overexpression in MCF-7 cells.

**H:** RNF181 depletion inhibited cell wound healing, which effect could be further rescued by RNF181 overexpression in MCF-7 cells.

**Supplementary Figure 2**

**A:** RNF181 depletion via siRNAs (DepMap data) could effective inhibit RNF181 mRNA level in MCF-7 cells.

**B:** RNF181 depletion via DepMap siRNAs could inhibit ERα protein levels in MCF-7 cells.

**C:** RNF181 depletion via DepMap siRNAs could inhibit ERα target gene levels in MCF-7 cells.

**D:** RNF181 depletion via DepMap siRNAs could inhibit ERE luciferase activity in MCF-7 cells.

**E:** RNF181 depletion via DepMap siRNAs could inhibit breast cancer cell proliferation in MCF-7 cells.

**F:** RNF181 depletion via DepMap siRNAs could inhibit wound-healing capacity in MCF-7 cells.

**G:** RNF181 depletion via DepMap siRNAs could inhibit clone formation capacity in MCF-7 cells.
