# Supplementary figures and images for: The ubiquitin ligase RNF181 stabilizes ERα and modulates breast cancer progression
Source: Oncogene. 2020 Sep 24;39(44):6776–88. doi: 10.1038/s41388-020-01464-z (PMC7605433; doi:10.1038/s41388-020-01464-z)

## Slide 1
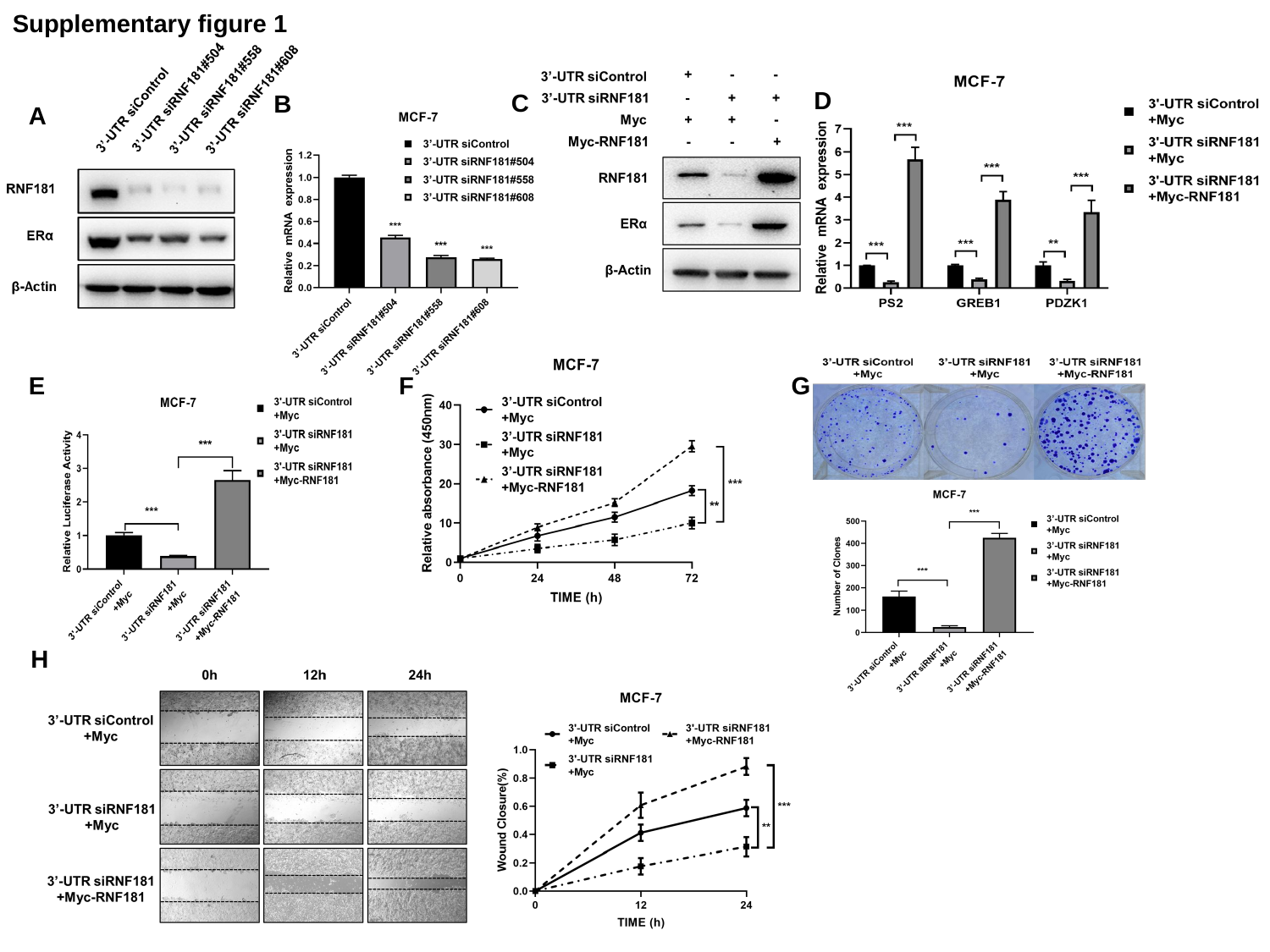

Supplementary figure 1
D
C
B
A
E
F
G
H

## Slide 2
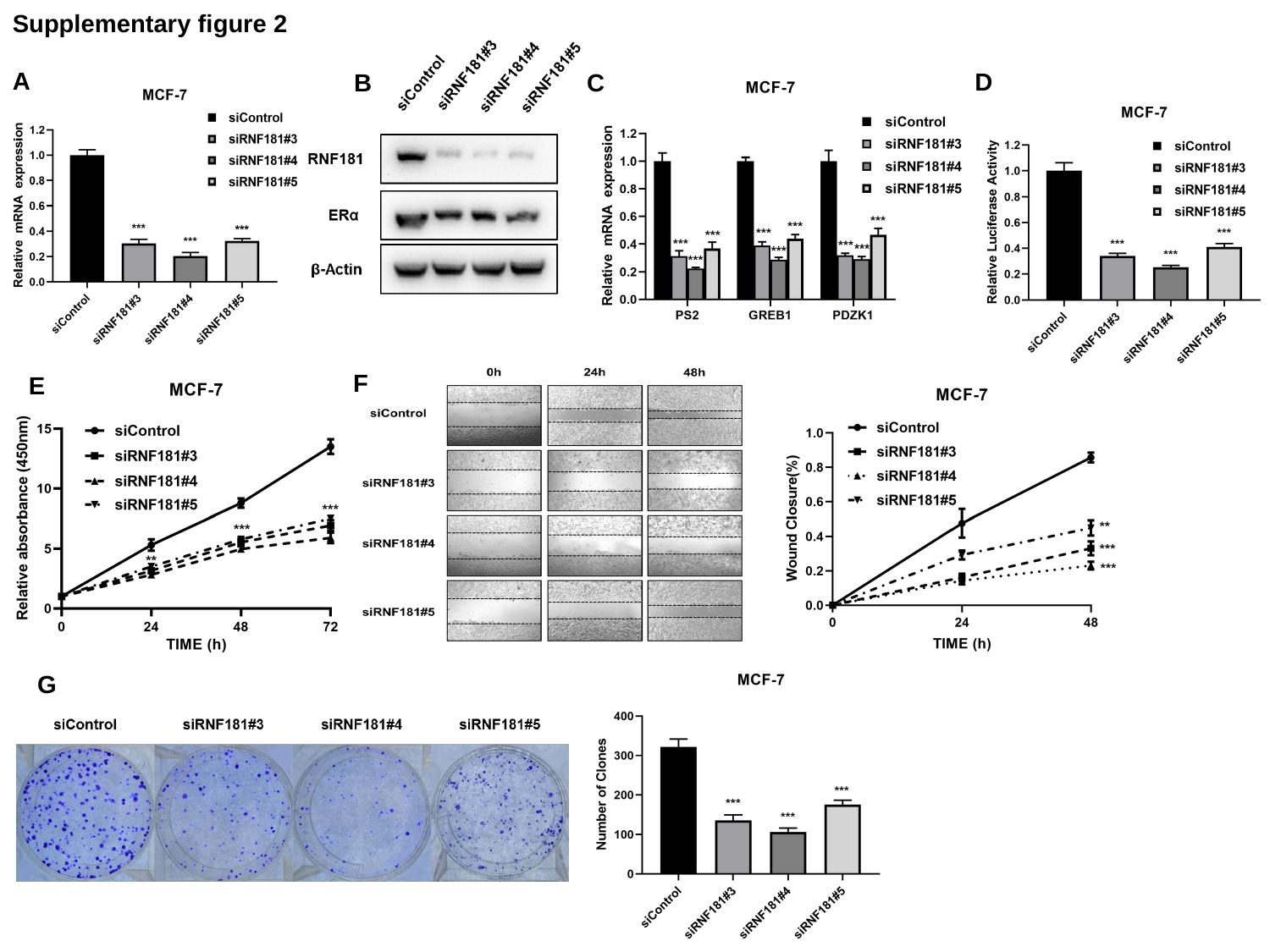

Supplementary figure 2
A
D
B
C
F
E
G

Supplement: Supplementary file 2 — Supplementary figures [file 41388_2020_1464_MOESM2_ESM.pptx]
