# Supplementary material for: The ubiquitin ligase RNF181 stabilizes ERα and modulates breast cancer progression
Source: Oncogene. 2020 Sep 24;39(44):6776–88. doi: 10.1038/s41388-020-01464-z (PMC7605433; doi:10.1038/s41388-020-01464-z)
Supplement: Supplementary file 3 — Supplementary tables [file 41388_2020_1464_MOESM3_ESM.pptx]

## Slide 1
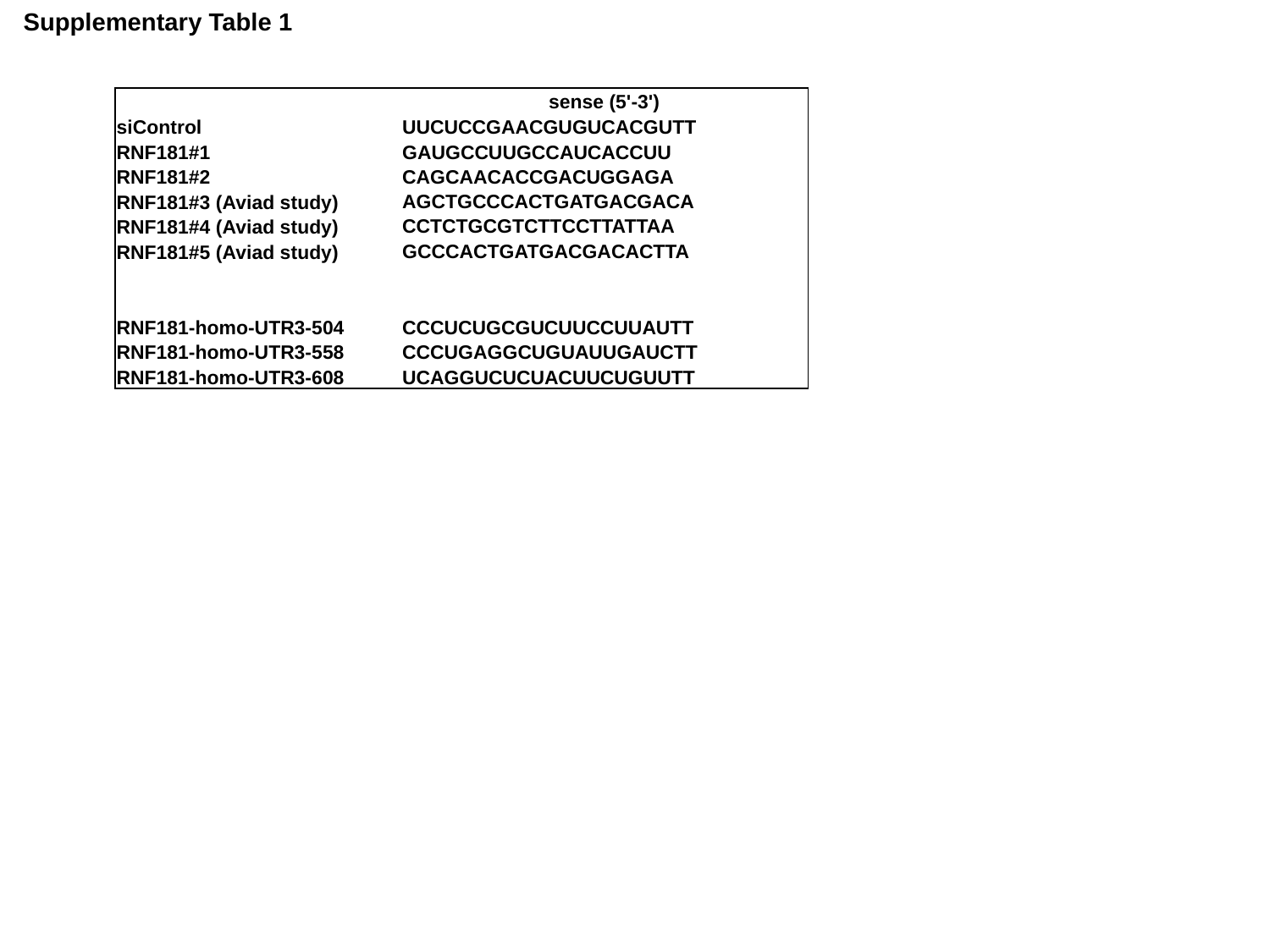

Supplementary Table 1
| | sense (5'-3') |
| --- | --- |
| siControl | UUCUCCGAACGUGUCACGUTT |
| RNF181#1 | GAUGCCUUGCCAUCACCUU |
| RNF181#2 | CAGCAACACCGACUGGAGA |
| RNF181#3 (Aviad study) | AGCTGCCCACTGATGACGACA |
| RNF181#4 (Aviad study) | CCTCTGCGTCTTCCTTATTAA |
| RNF181#5 (Aviad study) | GCCCACTGATGACGACACTTA |
| | |
| | |
| RNF181-homo-UTR3-504 | CCCUCUGCGUCUUCCUUAUTT |
| RNF181-homo-UTR3-558 | CCCUGAGGCUGUAUUGAUCTT |
| RNF181-homo-UTR3-608 | UCAGGUCUCUACUUCUGUUTT |
